# Maxillary reconstruction to enable implant insertion: a retrospective study of 181 patients

**DOI:** 10.1186/1746-160X-4-31

**Published:** 2008-12-16

**Authors:** Joël Ferri, Jean-Pascal Dujoncquoy, José Mario Carneiro, Gwénael Raoul

**Affiliations:** 1 Chairman of the Maxillo-Facial Department at Lille 2 University, Lille, France; 2 Senior Resident in Oral and Maxillo-Facial Surgery at Lille 2 University, Lille, France; 3 Formerly Resident of Dento-Maxillo-Facial Orthopaedics and Research Program at Lille 2 University, Lille, France; 4 Fellow of the Maxillo-Facial Department at Lille 2 University, Lille, France

## Abstract

**Background:**

The purpose of the present study was to evaluate different types of maxillary pre-prosthetic surgery using autogenous bone graft and suggest a guideline for maxillary reconstruction to place implant.

**Methods:**

181 patients (125 females and 56 males), age range from 16 to 76 years old, were operated at the Maxillo-Facial Service of the Lille's 2 Universitary Hospital Center (Chairman Pr Joël Ferri). Different techniques were used, but always with autogenous bone grafting. 21 patients underwent a Lefort 1 procedure, 139 underwent sinus graft with or without vestibular onlay graft and 21 underwent onlay graft. This surgical procedure was made to allow the insertion of 685 implants.

**Results:**

The patients were evaluated by clinical and radiological assessment. In the cases of Lefort 1, the rate of successful osteointegration was higher when the implants were placed in the second part of a two stages procedure: 92%, against 81% for one stage. In cases of sinus lift procedure, the rate of implant success was 98%. The infection rate was 3.5%. There was no significant resorption and the type of prosthesis used was a denture retained by a bar or fixed bridge. In cases of onlay graft, the implant insertion success was 97% and there was no infection. The amount of resorption was more significant in the pre-maxilla than in the other areas and the type of prosthesis used was fixed dentures.

**Conclusion:**

These observations demonstrate that: the aetiology of the bone defect indicate the type and number of the surgical procedures to re-established good jaws relationship and give the bone conditions to implant insertion successful.

**Clinical Relevance:**

A guideline for surgical decision in the maxillary reconstruction for oral rehabilitation by implants may help to prevent failures of osseous resorption disorders and to foresee the investment of the bone in quality and necessary quantity.

## Background

From a maxillo-facial surgeon's point of view, prosthetic rehabilitation associated with a facial balance remains the most important goal to choose a specific procedure in the challenge of maxillary reconstruction to facilitate implant insertion. Great advances have been made with autogenous bone to enable implant insertion in maxilla with or without structural and architectural defect [1,2]. So, well as several works in this domain, where there is major atrophy of the maxilla it's not easy to choose between an onlay grafting procedure and a Lefort 1 with graft. When choosing a Lefort 1 procedure, should we insert the implant at the same time as we perform the graft as described by Sailer, or should we adopt a two stage procedure as advocated by Cawood and Stoelinga [3,4]. As yet there is no clear answer to these questions. In cases of sinus lift or onlay grafting, the rate of resorption and the infection considerably vary on the different publications and techniques [5-7]. In this study, 181 retrospective cases for which different techniques were used (Lefort1, onlay grafting and sinus lifting, always with autogenous bone graft (skull, chin or iliac crest)) were analysed. We present our results and we propose a guideline for surgical decisions.

It is clear that biomaterials have their indications specially for sinus or alveolar sockets grafting. We often use them, however in order to compare the techniques of grafting and not the different materials which were grafted we decided to exclude the cases were biomaterials were used.

## Methods

Our sample consists of 181 patients, 125 females (69%) and 56 males (31%), with a total of 685 implants inserted, and all presented for maxillary reconstruction with autogenous bone graft to enable implant insertion. The age ranged from 16 to 75 years old and comprised the following aetiology of the bone defects: 22 patients (12.15%) had ectodermal dysplasia or congenital missing teeth, 21 (11.61%) patients had traumatology after-effect and 138 (76.24%) had periodontitis after-effect. The follow up was between 6 to 61 months.

### Above specific surgical procedure

181 cases were investigated for which different techniques were used: Lefort1, onlay grafting and sinus lifting, always with autogenous. Depending of the amount of the defect the bone came from the chin, the skull, or the the iliac crest. In case of small defect we used first the chin, in the other cases the choice was the skull or the iliac crest. The choice between iliac crest or skull was done depending of the patient request (some patient were afraid by a skull harvesting so we had to take the graft from the iliac crest) and the skull thickness (under 8 mm we harvest the bone from the iliac crest to avoid any risk of cerebral damage). 9 patients (4.97%) underwent Lefort 1 one-step procedure and 68 implants (9.93%) were inserted, 12 patients (6.63%) underwent Lefort 1 two-step procedure and 95 implants (13.87%) were inserted; 139 patients (76.80%) underwent 199 sinus lift only and 481 implants (70.22%) were inserted; 21 patients (11.6%) underwent onlay grafting with a formwork technique and 41 implants (5.98%) were inserted. In cases of Lefort 1, the implants were inserted at the same time with the technique inlay one-step procedure (IOSP), according to Sailer's technique, or as a second delayed operation inlay two-step procedure (ITSP), according to Cawood-Stoelinga's technique [3,4].

**Figure 1:**
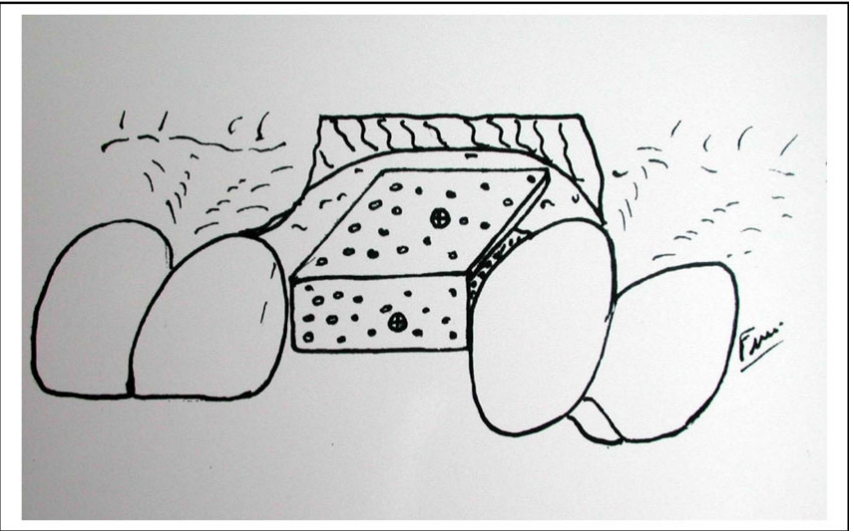
The framework technique: By means of mini bone plates we build a framework, which gives a compression to the bone particles. The bone plates are perforated then fixed with screws which will be removed when the implants are inserted.

The Sailer's or Cawood-Stoelinga's technique consists in performing a Lefort 1 osteotomy of the maxilla. The section must be done with care in order to avoid any damage of the nasal mucosa or fracture of the maxilla which is very fragile in some cases. Then the sinus membrane is carefully but completely removed from the sinus floor. The sinus floor is then filled with the bony graft and the implants are inserted in the new reconstructed bone. The maxilla is then fixed in a new position according to the prosthodontic planning. The onlay one difference between Sailer's and Cawood-Stoeleinga's procedure is the timing of the implants insertion. In cases of sinus grafting, we used Tulasne's technique. For the onlay grafting of alveolar areas involving until 4 teeth, the technique used was a framework which avoids any compression by the soft tissues. This technique itself consists (by means of a bone miniplates which has been perforated to allow blood supply from the soft tissues) to build a framework. Then the defect is filled with the bone shaving, the structure is established by screws (figure 1).

Whatever the procedure used it was clinically evident that the amount of bony reconstruction, if stable was sufficient to insert the implants.

### Performance appraisal

The results were evaluated by clinical and radiological assessment by employee of Denta CT Somatom Plus Siemens. The Denta CT evaluation was carried out just before the surgery and at the end of the fifth month. The height and thickness of the bone was measured by calculating the average of the molar and canine sites in cases of Lefort 1. In cases of onlay grafting on sinus floor or alveolar area, we averaged the different sites where the implants would be inserted. Pains, mobility, radiological hypodensity around the implant, non usable position of the implant, were considered as implant failures.

The prosthetic rehabilitation is described in relation to the type of preprosthetic surgical procedure.

## Results

The results are given by the tables 1 and 2.

**Table 1:** The average gain of the grafting procedures in different areas.

**Type of Surgery**	**Lefort 1 one-step procedure (IOSP)**				**Lefort 1 two-step procedure (ITSP)**				**Sinus lift**		**Onlay grafting**	
**Bone Area**	Molar		Canine		Molar		Canine					
				
**Bone Quantity**	Height	Thick ness	Height	Thick ness	Height	Thick ness	Height	Thick ness	Height	Thick ness	Height	Thick ness

**Before Surgery (mm)**	3	4	6	3	4	2.5	5	2.5	3	3	4	2

**After Surgery (mm)**	15	10	12	11	14	13.5	12	10.5	9.5	10	10	7

This table give the bone increment in different areas.

**Table 2:** Result of implant rate success, kind of prosthesis realised, and infection rate.

**Surgical Procedures**	**Number of patients**	**% of rate of implant success**	**Prosthetic rehabilitation**	**Graft Infection rate**	**Special Remarks**
**Lefort 1 One-step procedure IOSP**	9	81%	1 fixed denture 8 retained dentures	0%	Sometimes poor quality of bone

**Lefort 1 Two-step procedure ITSP**	12	91%	11 fixed dentures 1 retained denture	0%	Sometimes poor quality of bone

**Sinus grafting**	139 patients (199 sinus)	98%	199 fixed dentures	3.5%	High quality of bone

**Onlay grafting**	21	97.5%	21 fixed dentures	0%	Sometimes important resorption High quality of bone

This table link the implant insertion success and the number of patients with all clinical and surgical procedures with special remarks about bone quality and resorption.

The table 1 gives the average gain of the grafting procedures in different areas.

In case of Lefort 1 the measurements were done in the molar and canine area. In case of the other procedures the measurement were done on the site of the surgery. The study was done in two directions vertically (height) and transversaly (thickness).

The gain was (13–15) 12 mm height and (4–10) 6 mm thick,(12-6) 6 mm height and (11-3) 8 mm thick in molar and canine area respectively, with IOSP procedure.

The gain was (14-4) 10 mm height and (13.5-2.5) 11 mm thick, (12-5) 7 mm height and (10.5-2.5) 8 mm thick in molar and canine respectively, with ITSP procedure.

In the sinus lift procedure the benefit was (9.5-3) 6.5 mm vertically, and (10-3) 7 mm transversally.

In the case of "simple" onlay procedure the gain was (10-4) 6 mm vertically and (7-2) 5 mm transversally.

The table 2 presents the result of implant rate success, the different kind of prosthesis realised, the infection rate, and the number of patients in each procedure.

The vast majority of procedure is the sinus grafting; the quality of the bone was appreciated subjectively and reported when it seemed poor.

In case of IOSP 68 implants were inserted and 55 were osteointegrated, (81%). 8 patients had removal denture (88.89%).

In case of ITSP 95 implants were inserted and 86 were osteointegrated (91%). 11 patients had fixed dentures (91.6%).

In case of sinus grafting procedure 481 implants were inserted and 471 were osteointegrated (98%).

In case of onlay grafting procedure 41 implants were inserted and 31 were osteointegrated (97.5%).

## Discussion

The increment of the bone mass is clear. Each technique improves the implant insertion. Thus, we can conclude that the different procedures can all be used to enable implant insertion.

### The rate of success for osteointegration

With the Lefort 1 procedure, the success rate of integration appears less important in cases of IOSP compared with ITSP (81% versus 91%). This point is well known and already mentioned in literature [8,9].

It can be explained by the difficulty in assessing the future bone resorption area. Some implants could have been fixed in the site where bone will resorb.

In the IOSP it is clear that the first goal before any other consideration was to place the implants that would optimally stabilise the graft. Consequently the position of the implant was not always in the best place from a strictly prosthetic point of view. All these factors explain a lower rate of success in the one-stage procedure compared with a two-stage one.

We have not done investigation into the quality of the graft but it seemed to us that there is no significant difference in the quality between IOSP and ITSP. Of course this is just a subjective feeling without any scientific datas. But when we look at the CT scan at the 5^th ^month it seemed to us that there were no difference between the two procedures concerning the graft density, and a high density is often correlated with a good quality. Many factors are necessary to provide a "good" bone for implantology, but the high density is an important point because it provides a good chance of primary stability.

**Figure 2:**
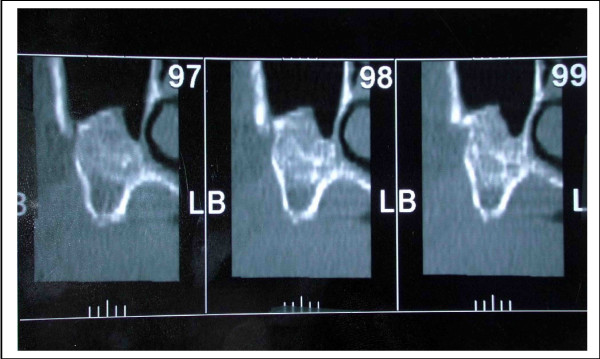
Density of the graft in a sinus grafting procedure: one can look that density can be higher in the graft than in the initial alveolar bone.

In the group of patients who had sinus graft with or without onlay, the osteointegration rate was 98%. This good result can be explained because of the two-step procedure which inserts the implant in a position taking into account the bone situation, and also because of the good density of the bone reconstruction. The average height was 9.5 mm, but the density was high. Sometimes, this density was higher than the initial alveolar bone itself (figure 2). This result confirms the high quality of this reconstruction which gives a rate of osteointegration similar to that of a normal bone [10].

In the group of onlay grafting procedure the results are the same as in the sinus lift one (97.5% and 98% of osteointegration).

These good results confirm the high quality of the bone reconstruction provided by this procedure.

### Bone resorption, quality and quantity of the graft, Lefort 1 with IOSP or ITSP

Despite a lack of precise evaluation we think that our insufficient bony results are due to resorption because the amount of bone we provided during the surgery was clinically important in all cases with sometimes an over correction. So the bone loss at the 6th month is due to resorption and not to a lack of initial reconstruction.

It seems to us that bone resorption varies a lot according to the location of the graft. The most dramatic losses were in the premaxilla. The amount of resorption is difficult to quantify, but undoubtedly it is in this area that the resorption was the most important. In some extreme cases we lost about 90% of the graft. The resorption seemed important in the anterior-posterior direction and vertically too. Such resorption with iliac crest bone has been reported [5].

**Figure 3:**
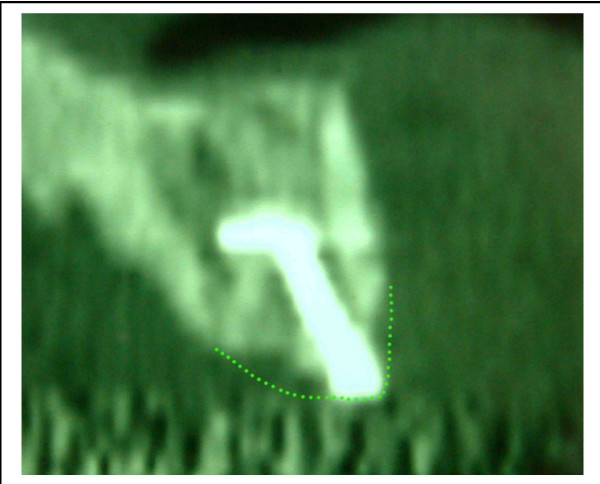
Resorption in the premaxilla: The bone loss is important in this area. The dotted line shows the initial position of the graft. One looks has a significant bone resorption.

**Figure 4:**
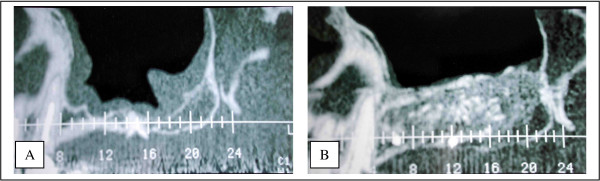
Improvement of the quality and quantity of the graft in a sinus grafting procedure. 4A – before. 4B – after

**Figure 5:**
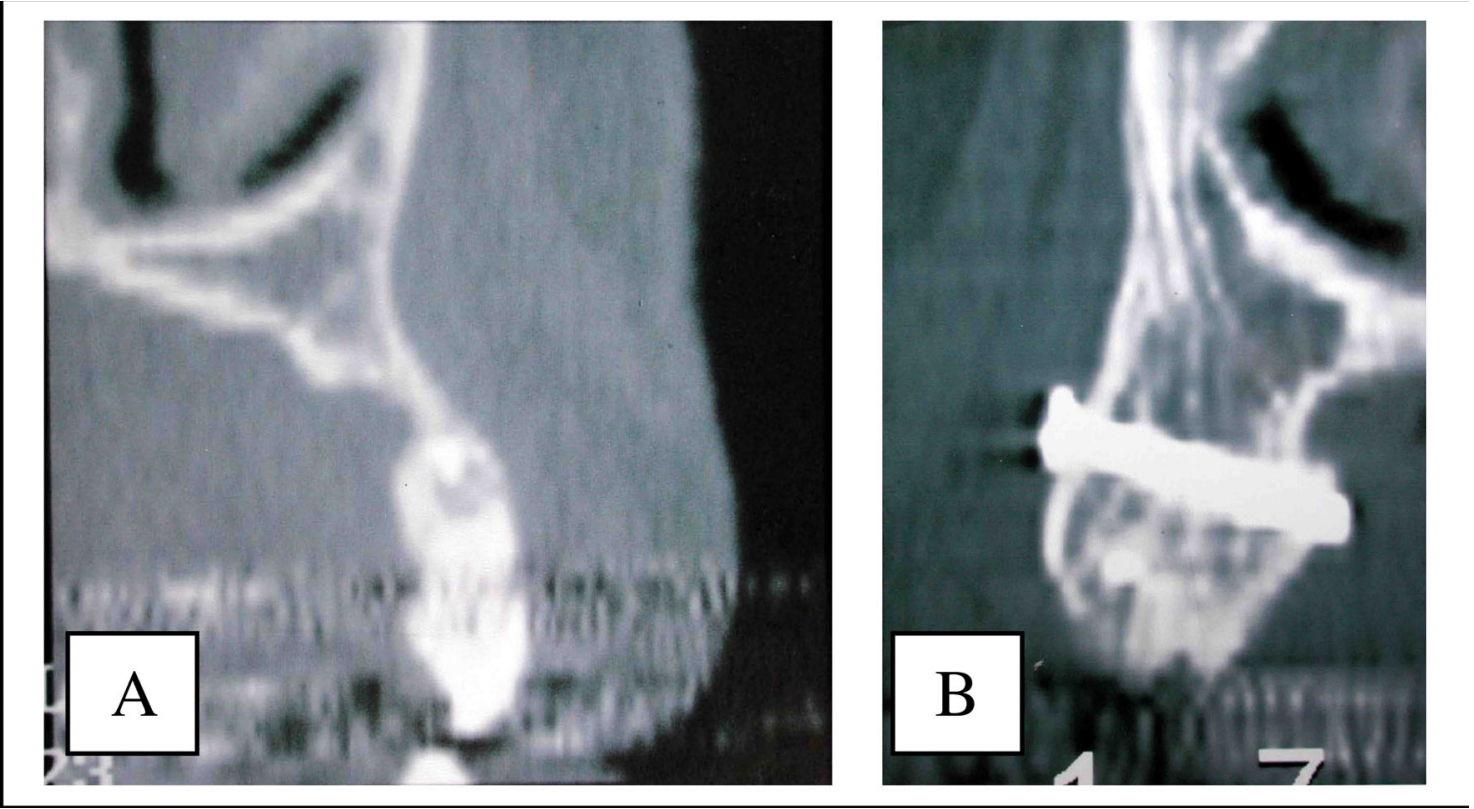
Improvement of the quantity and the quality of the bone after onlay grafting. In the premaxilla with a framework procedure. 5A – before. 5B – after. We build a bone framework which is filled with bone particles. The plates protect the graft against the soft tissues pressure. It also provides a high density to the reconstruction.

For us, the pathophysiology of the resorption was purely local. It explains the different results one can have in the same patient between the sinus and the premaxilla. There is no pressure from the soft tissues in the sinus compared with the premaxilla. The results are much better in the sinus (figures 3 and 4). In our opinion, one of the factors which induce the resorption is the pressure of the soft tissues. The origin of the graft seems to play an important role too [11]. The framework technique is used to protect the bone from this pressure. It is the membrane concept developed by many authors [12,13]. According to us, the building of a complete "framework", particularly in the vertical dimension, reduces the resorption. However, it is sometimes insufficient to avoid it completely (figure 5).

In cases of major premaxilla atrophy, we do not recommend an onlay grafting procedure for two reasons:

1- After grafting, in case of resorption, the jaw relationship could have an adverse effect on the prosthetic rehabilitation even if there is still enough bone to insert the implant.

2- The treatment of the jaw discrepancy needs a large amount of bone. Thus, the resorption risk is higher.

**Figure 6:**
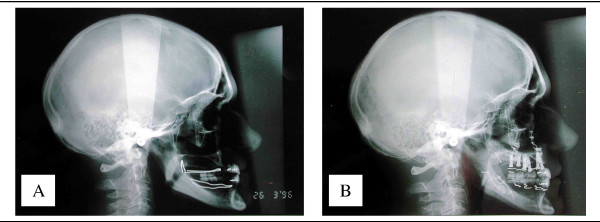
Advantages of the Lefort 1 procedure in case of jaw discrepancy: It improves jaw relationships. 6A – before. 6B – after.

**Figure 7:**
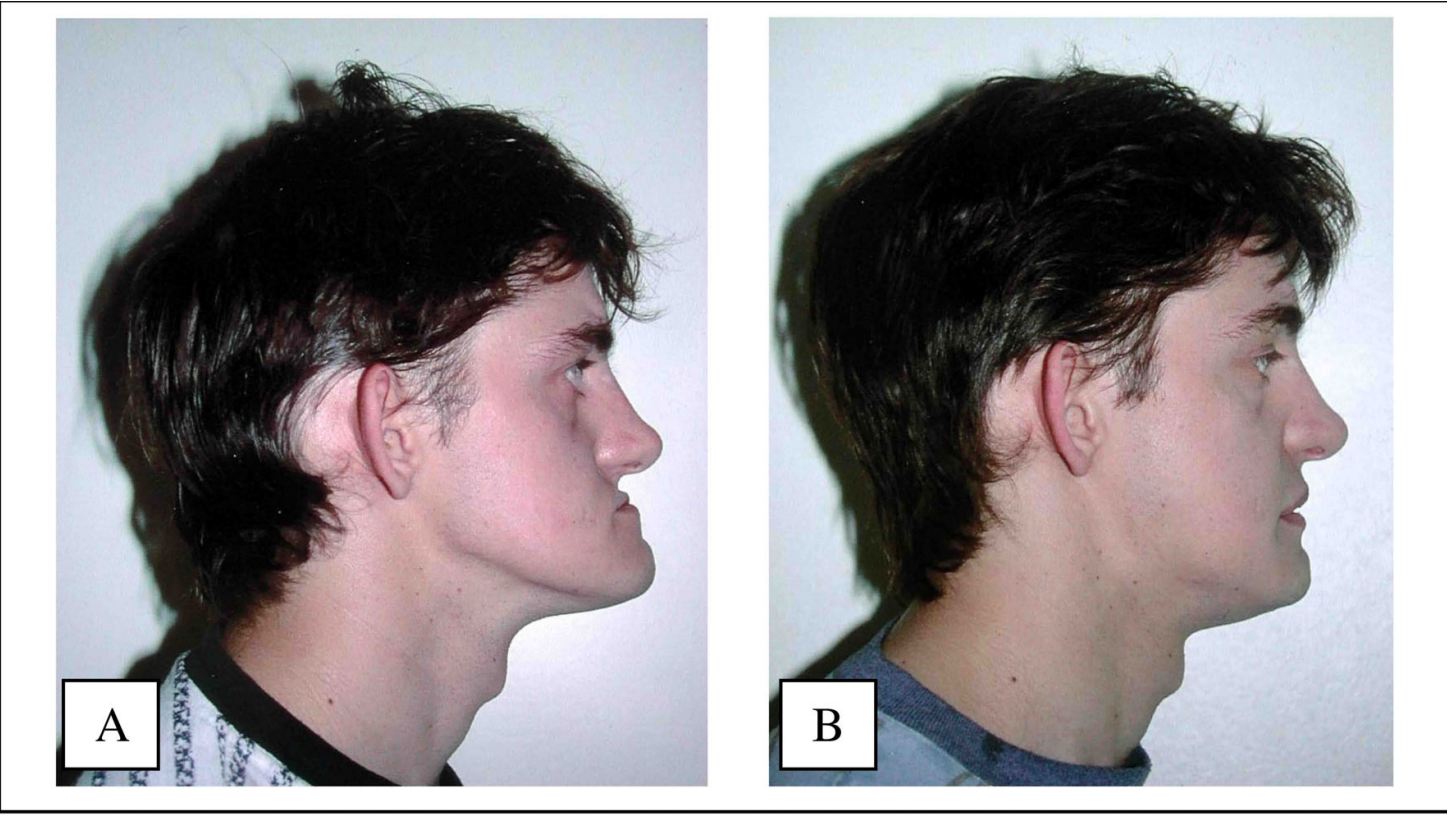
Advantages of the Lefort 1 procedure in case of jaw discrepancy: It improves facial appearance. 7A – before. 7B – after.

**Figure 8:**
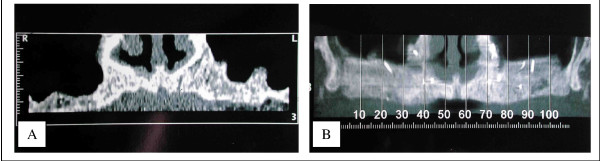
Advantages of the Lefort 1 procedure in case of jaw discrepancy: It provides bone where needed. 8A – CT scan before. 8B – CT scan after.

Where there has been significant maxillary resorption, in which the maxillary balance (class III) makes prosthetic rehabilitation difficult, it is better to perform a Lefort 1. It has many advantages (figures 6, 7, 8):

1 – It solves, in one surgical procedure, the bone deficiency and the discrepancy of the jaws.

2 – It improves jaw relationships, simplifying prosthetic rehabilitation.

3 – The grafts are placed in ideal or near ideal locations.

4 – It improves facial aesthetics by inducing a younger appearance.

However, it remains difficult to choose between a one or two-stage procedure. In our study, it is clear that the two-step procedure gives a better osteointegration rate. Different reasons could account for these results:

1 – In ITSP, the implant insertion is more accurate than in a one-step procedure, where it is always difficult to find the best position for graft fixation, as well as for prosthetic rehabilitation. In some cases the osteointegration was perfect, but the implant could not be used to support the prosthesis. This was considered as an implant failure.

2 – If the bone resorption occurs at the site where an implant was inserted, there is inevitably an implant loss.

3 – The surgical procedure is difficult, especially where the sinus floor is very thin [14]. In such cases, it is difficult to achieve complete immobility of the implant. This instability may induce non-osteointegration.

Nevertheless, the one-step procedure still has some indications:

1 – When patients do not want to undergo a second surgical procedure.

2 – In cases where the accuracy of the implant settlement is not essential.

**Figure 9:**
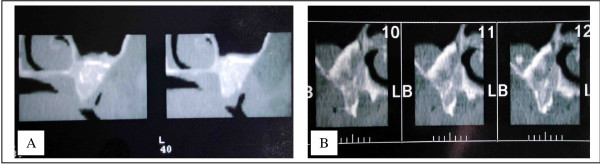
Density of the bone after a sinus grafting procedure compared with a Lefort 1 technique. The density is sometimes less important with the Lefort 1 than in a sinus lift as we can see here. 9A – Sinus grafting. 9B – Lefort 1.

In Lefort 1 procedures, it appears that there was no quantitative resorption. There were no significant differences between IOSP and ITSP in the density and the quantity of the graft. The amount of bone was always sufficient but the density was sometimes poor. Bone density was always less compared to that following a sinus grafting procedure (figure 9). However, we did not know the initial radiological density of the graft, so it is difficult to investigate the qualitative resorption process itself. This poor density can be explained because of a poor vascular supply. Vascularization of the sinus floor is poor and it could be insufficient to ensure adequate blood supply for graft viability. This hypothesis is also supported by the fact that the Lefort 1 reduces the vascularization (section of the naso-palatine pedicle). The second hypothesis is an initial lack in density of the graft. It could be explained by a general osteoporosis which seems to have a higher rate in patient with periodontitis (the most important aetiology of tooth loss in our series) [15]. This osteoporosis produces poor trabecular bone with a low bone density.

### Graft infections

In cases of onlay grafting, infections occurred because of secondary failure of the sutures which resulted in exposure of the graft exposure in the mouth. This complication never appeared in our presented series, but it occurred in other patients. The final result was always good with the removal of the few exposed bone particles. Then healing was spontaneously perfect. The aetiology is much more difficult to understand with respect to the sinus. In some cases there was a sinus membrane perforation, but in others, there was none. Our rate of infection was 3.5%. It seems less important with experience, which is in favour of sinus membrane perforation aetiology. We had no infections in cases of Lefort 1.

### Prosthetic rehabilitation

Different kinds of prosthetic rehabilitation, related to the different surgical procedures, have been used.

Our results show a retained removal over-denture in most cases of Lefort 1 procedures with IOSP (88.89%). This technique of rehabilitation was used because it was easier in cases of non ideal emerging position of the implant. It is the best prosthetic procedure in such cases [16]. In cases of ITSP we did a fixed denture in 91.6% of cases. This kind of rehabilitation was chosen because of a very accurate location of the implants. We can conclude that Lefort 1 with IOSP gave less accurate implant insertion, and this way provided an over denture prosthetic rehabilitation more frequently.

## Conclusion

**Figure 10:**
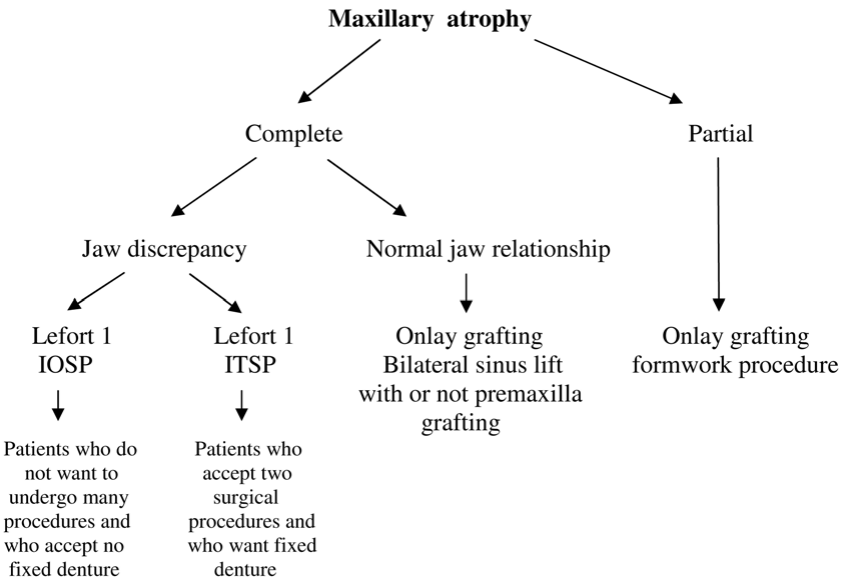
This figure gives a guideline for maxillary reconstruction prior to implant insertion.

From this study, we have been able to develop a guideline for maxillary reconstruction prior to implant insertion (figure 10).

The first point to take into account is the maxillary status regarding the teeth. Two cases can be faced, partial edentulous maxillae and total edentulous one.

In case of partial edentulous maxilla the graft will be done in an onlay manner; it will provide an excellent result in most of cases. The sinus grafting will be done if necessary in case of deficit in this area.

These techniques provide excellent results despite the fact that some resorption can arise. The reasons of these resorptions are not clear at the moment. Some infections can also arise in sinus floor grafting.

In cases of total edentulous maxilla, jaws relationship must be evaluated. In cases of normal jaw relationship, we recommend to perform onlay and sinus lift grafting. Of course the area of grafting depends of the defect site. If jaw discrepancy is present, it must be corrected with a Lefort 1 procedure. One time procedure provides bone and implants in a single time, however the implant rate success is lower than a two steps procedure. In most of cases the denture will be a non fixed one. This procedure must be proposed to patients who do not want to undergo many procedures and who accept non fixed denture. Two steps procedures must be proposed to patients who do not want to undergo many surgeries and who accept no fixed denture.

## Consent

Written consent for publication of the pictures used in figure 7 was obtained from the patient. A copy of the written consent is available for review by the Editor-in-Chief of this journal.

## Competing interests

The authors declare that they have no competing interests.

## Authors' contributions

JF operated 181 included patients in this study. JPD and JMC wrote this article. The original idea for this work comes from GR who also acted as a supervisor.
